# Managing a Patient With Hypertensive Crisis

**DOI:** 10.7759/cureus.90003

**Published:** 2025-08-13

**Authors:** Jalal Ibrahim, Torrin P Jacobsen, Adina Boulos, Jason S DeFrancisis, Prathap Simhadri

**Affiliations:** 1 Dermatology, Lake Erie College of Osteopathic Medicine, Bradenton, USA; 2 Radiology, Lake Erie College of Osteopathic Medicine, Bradenton, USA; 3 Psychiatry, Lake Erie College of Osteopathic Medicine, Bradenton, USA; 4 Orthopedic Surgery, Lake Erie College of Osteopathic Medicine, Bradenton, USA; 5 Internal Medicine/Nephrology, Advent Health, Florida State University (FSU) College of Medicine, Daytona Beach, USA

**Keywords:** acute kidney injury, clinical decision-making, diagnostic imaging, flash pulmonary edema, hypertensive emergency, renal artery stenosis

## Abstract

Renal artery stenosis (RAS) is a common yet under-recognized cause of secondary hypertension and acute kidney injury, especially in elderly patients with multiple comorbidities. We report the case of a 74-year-old woman with longstanding hypertension, chronic kidney disease stage 3, and tobacco use who presented with flash pulmonary edema and hypertensive emergency. Despite a negative renal duplex ultrasound, persistent clinical suspicion prompted further evaluation with renal angiography, which revealed significant right RAS. Following successful stenting, the patient showed marked improvement in renal function and was discharged with stable kidney parameters. This case highlights the importance of integrating clinical judgment, physical exam findings, and laboratory data when noninvasive imaging is inconclusive. It also emphasizes the value of timely intervention in improving patient outcomes and the need to rely on a comprehensive diagnostic approach in high-risk patients.

## Introduction

Hypertensive emergency (HE) is defined as blood pressure above 180 mmHg systolic and 120 mmHg diastolic, with signs of acute target organ damage, such as flash pulmonary edema or kidney injury [1]. Some findings indicate HE is stroke (38% of cases), followed by pulmonary edema (35%), as seen in this patient, then coronary syndromes (25%) [1]. The key difference between a HE and hypertensive urgency is solely based on the signs of acute target organ damage involved with the patient; if no signs are observed, then it is classified as hypertensive urgency. Related pathologies inducing secondary hypertension, such as hyperaldosteronism, pheochromocytoma, nephritic syndrome, and glucocorticoid use, must be considered. These differentials should be ruled out concurrently before confirming the diagnosis of renal artery stenosis (RAS). This manuscript presents a case involving life-threatening RAS symptomatology in a patient with multiple comorbidities. These comorbidities created difficulty with imaging studies. With that being said, the clinical examination, laboratory diagnostics, and alternative studies helped reveal their established and relevant importance.

## Case presentation

We present the case of a 74-year-old Caucasian female with a significant medical history of long-standing hypertension, tobacco use, and chronic kidney disease (CKD) stage 3 with a historical baseline creatinine of 1.3 mg/dl, who presented with complaints of shortness of breath for two days. Notably, she had undergone a left carotid endarterectomy in 2017. The patient’s vital signs indicated blood pressure above 220/130 mmHg with signs of pulmonary edema and congestive heart failure. The patient's medication compliance was poor, as she would not take her medications consistently. She was prescribed amlodipine, metoprolol, and hydralazine for her blood pressure. Additional vital signs showed a temperature of 98.2°F, a respiratory rate of 28/min, a heart rate above 100 bpm, and 79% oxygen saturation on room air. As per clinical guidelines, the patient was classified as experiencing HE and admitted for treatment. Upon initial laboratory analysis, the patient’s laboratory results were hemoglobin 10.3 g/dl, troponin 26 ng/mL, and proBNP of 6322 pg/mL (Table 1).

**Table 1:** Summary of lab results, including CBC, CMP, chest X-ray, and renal ultrasound WBC: white blood cells, RBC: red blood cells, Hgb: hemoglobin, Hct: hematocrit, MCV: mean corpuscular volume, RDW: red cell distribution width, CMP: complete blood count, BUN: blood urea nitrogen, Cr: creatinine, ALP: alkaline phosphatase, AST: aspartate aminotransferase, ALT: alanine aminotransferase, CHF: congestive heart failure, CKD: chronic kidney disease

Test	Result	Normal range	Notes
CBC			
WBC	11.5 x10³/μL	4.0-10.5 x10³/μL	Elevated
RBC	3.87 x10⁶/μL	4.7-6.1 x10⁶/μL	Low
Hgb	10.3 g/dL	13.5-17.5 g/dL	Low
Hct	32.1%	38.8-50.0%	Low
MCV	82.9 fL	80-100 fL	Normal
RDW	17.4%	11.5-14.5%	Elevated
Platelets	275 x10³/μL	150-400 x10³/μL	Normal
CMP			
Sodium	137 mmol/L	135-145 mmol/L	Normal
Potassium	4.4 mmol/L	3.5-5.0 mmol/L	Normal
Chloride	101 mmol/L	98-107 mmol/L	Normal
CO2	23 mmol/L	22-30 mmol/L	Normal
Glucose	159 mg/dL	70-100 mg/dL (fasting)	Elevated
BUN	44 mg/dL	7-20 mg/dL	Elevated
Cr	1.75 mg/dL	0.6-1.2 mg/dL	Elevated
Calcium	9.3 mg/dL	8.5-10.2 mg/dL	Normal
Total protein	7.4 g/dL	6.0-8.5 g/dL	Normal
Albumin	4.3 g/dL	3.5-5.0 g/dL	Normal
Bilirubin (total)	0.6 mg/dL	0.1-1.2 mg/dL	Normal
ALP	91 U/L	44-121 U/L	Normal
AST	14 U/L	10-40 U/L	Normal
ALT	10 U/L	7-56 U/L	Normal
BUN/Cr ratio	25	10:1-20:1	Elevated
Anion gap	13	8-16	Normal
Imaging			
Chest radiograph	CHF/fluid overload	-	Diffuse interstitial edema and small effusions with bibasilar atelectasis
Comparative renal ultrasound (1 month before admission)	Atrophic left kidney with cortical thinning	-	Ultrasound was done to confirm the diagnosis of CKD stage III B

During her hospitalization, her serum creatinine rose to 1.75 mg/dL, suggesting an acute decline in renal function. Shown below is the rise in the patient's creatinine, blood urea nitrogen (BUN), and glomerular filtration rate (GFR) levels throughout her stay (Table 2).

**Table 2:** Patient's renal function parameters, including creatinine (mg/dL), BUN, and estimated GFR over a six-day period. The data show a progressive decline in kidney function, with worsening creatinine, BUN, and GFR values over the first four days (the fourth day is when the stent was placed), followed by a notable improvement in renal function on days five and six BUN: blood urea nitrogen, GFR: glomerular filtration rate

Day	Creatinine (mg/dL) (normal: 0.6 -1.2)	BUN (mg/dL) (normal: 7-20)	GFR (mL/min/1.73m²) (normal: ≥90)
Initial	1.75	44	29
1 day after	2.18	55	22
2 days after	3.4	70	13
3 days after	4.6	83	9
4 days after	4.9	94	8
5 days after	2.4	73	19
6 days after	1.4	44	38

**Figure 1:**
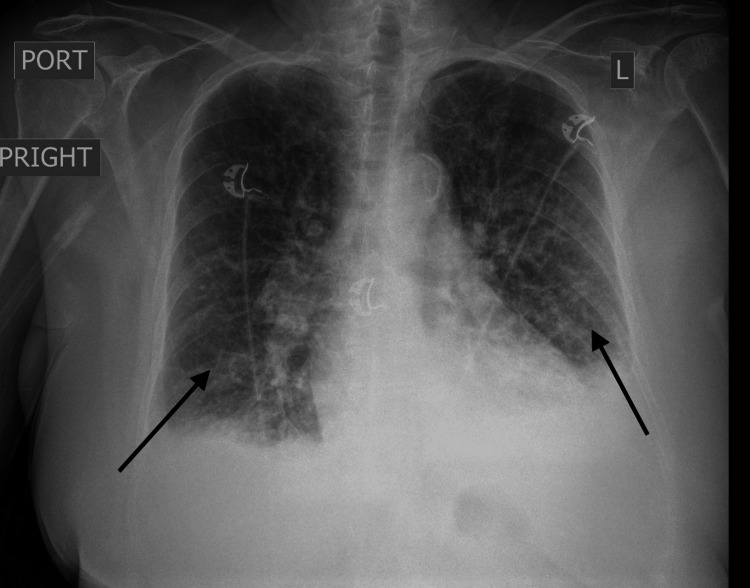
Chest X-ray demonstrating flash pulmonary edema with bilateral pleural effusions; arrows highlight key areas of pulmonary congestion and fluid accumulation

**Figure 2:**
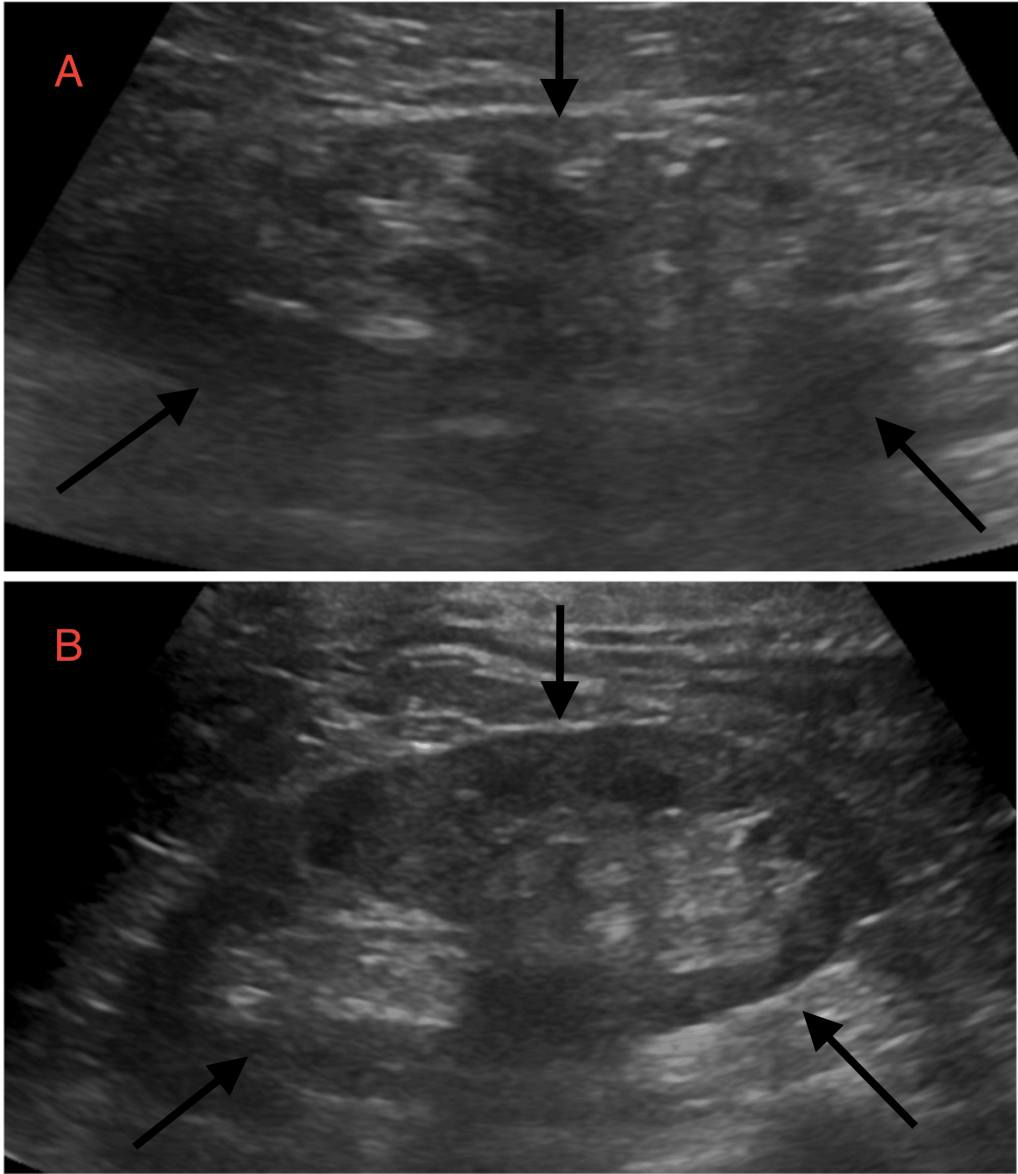
Renal ultrasound revealed a left kidney measuring 9.1 × 3.6 × 4.3 cm (A) and a right kidney measuring 10.7 × 1.0 × 1.9 cm (B). Although sonographic findings were unremarkable for RAS, the left kidney appeared mildly atrophic. Arrows indicate the dimensions and surrounding anatomical context of each kidney RAS: renal artery stenosis

**Figure 3:**
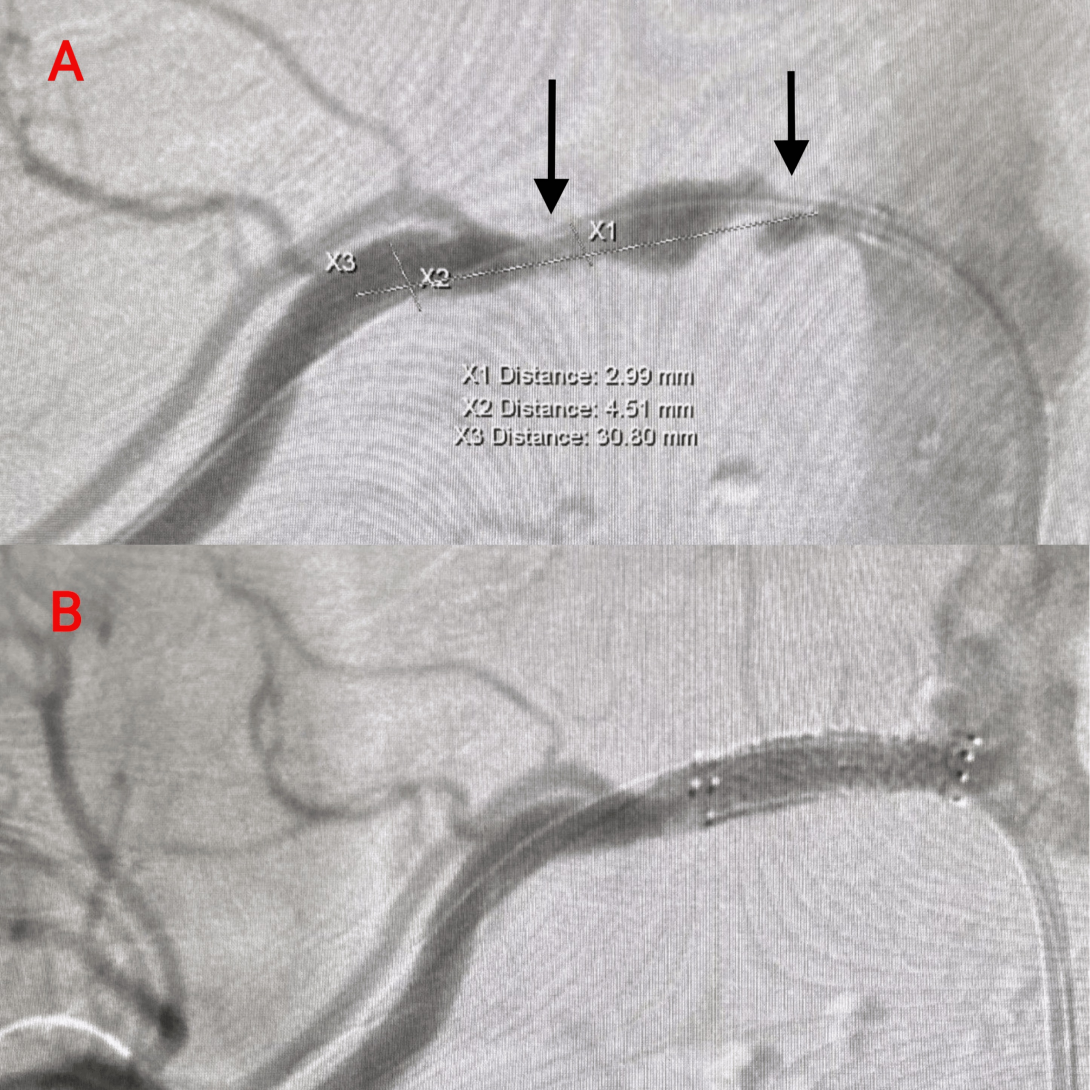
(A) Renal angiogram of the right renal artery demonstrating two significant stenoses. The rightmost arrow marks the most severe and tortuous narrowing proximal to the ostium, with an estimated diameter of <2.5 mm (unmeasurable). A second measurable stenotic lesion, located distal to the ostium at X1, measures 2.99 mm in diameter. X2 denotes the diameter of a normal, non-stenotic reference vessel segment, while X3 indicates the estimated stent length considered for the procedure. (B) Post-stent deployment angiogram demonstrating successful revascularization of the right renal artery, with restoration of vessel caliber and no evidence of residual stenosis

Given her history of poorly controlled hypertension and acute kidney injury (AKI) on top of CKD, nephrology was consulted. She was diagnosed with a combination of atherosclerotic RAS, contributing to AKI, flash pulmonary edema, and HE (Figure 1). She was put on amlodipine, hydralazine, metoprolol, and nitroglycerin to control her blood pressure. Other medications she was put on were aspirin, atorvastatin, and furosemide. Initial imaging with renal duplex ultrasound, however, did not demonstrate evidence of RAS (Figure 2). Due to persistent clinical concern, the patient was further evaluated with a renal angiogram, which revealed significant stenosis of the right renal artery (Figure 3). On day 4, the patient underwent successful stenting of the right renal artery, after which she experienced a marked improvement in urine output, and her renal parameters returned to near-baseline levels (Figure 3). At discharge (day 6), her creatinine improved to 1.4 mg/dL, and her BUN was 44 mg/dL. The immediate post-procedural improvement in renal function was striking, underscoring the importance of timely intervention in cases of atherosclerotic RAS, particularly when clinical suspicion remains high despite negative noninvasive imaging.

Four years after the diagnosis and successful stent placement, the patient is thriving. She remains off dialysis, with stable renal function and a good quality of life. This case illustrates the often-overlooked role of RAS in exacerbating hypertensive emergencies and AKI in elderly patients, especially those with a history of chronic hypertension. It also emphasizes the importance of continued diagnostic evaluation when initial imaging fails to identify the underlying cause and highlights the potential for significant recovery following appropriate intervention.

## Discussion

RAS is the narrowing of the renal artery, causing impaired blood flow to the kidney with subsequent downstream effects in the urinary tract. Atherosclerosis is the most common cause of RAS, and fibromuscular dysplasia (FMD) is the second most common cause of RAS [2]. Atherosclerosis constitutes roughly 60-90% of RAS cases and has been shown to more commonly affect males over the age of 45 years. It can occur as an isolated local lesion or involve the aorta proximal to the main renal artery [2,3]. Common risk factors for atherosclerosis include dyslipidemia, smoking, infection, inflammatory disease, diabetes mellitus, diet, family history, and age [4]. FMD constitutes 10-30% of cases and is primarily in women younger than 50 years of age, often involving middle, distal, or intrarenal artery branches [2,3]. Less than 10% of the remaining causes of RAS can be attributed to vasculitis, thromboembolic disease, or aorta-related aneurysm and dissection within a distance to the infrarenal region [2,3]. RAS can occur unilaterally or bilaterally and is associated with CKD and end-stage renal disease (ESRD) long-term [2,3]. RAS can be related to clinical syndromes, including ischemic nephropathy, hypertension, and cardiac syndromes; however, the diagnosis of RAS may be incidental in asymptomatic patients [5]. In symptomatic populations, an initial common presentation of RAS is unexplained resistant hypertension in patients where it would be otherwise unexpected [6]. As shown in our case presented, HE was expressed.

RAS can mimic several other conditions; therefore, performing an accurate patient history, physical evaluation, and laboratory diagnostics, and understanding similar pathologies are crucial to arriving at the correct diagnosis. Key conditions that may present similarly include hyperaldosteronism, pheochromocytoma, acute nephritic syndrome, and glucocorticoid-induced hypertensive emergencies. Differential diagnoses include the following:

Hyperaldosteronism typically features low renin levels, in contrast to RAS, which shows elevated renin [7]. Patients with RAS usually have normal serum sodium levels, whereas those with hyperaldosteronism often present with hypernatremia [7]. Hyperaldosteronism arises from excessive aldosterone production due to overactive adrenal glands, leading to increased blood pressure. Unlike RAS, which is caused by renal artery narrowing, hyperaldosteronism involves the adrenal glands and is related to hyperplasia of the glands themselves. When confirming hyperaldosteronism, it’s vital to consider the impact of various medications. Medications such as antihypertensives, antihistamines, antidepressants, estrogen, or NSAIDs can alter the aldosterone/renin ratio and thus must be taken into consideration [7].

Pheochromocytoma and RAS can cause elevated blood pressure, but they originate from different mechanisms. Pheochromocytoma arises from chromaffin cells in the adrenal medulla, resulting in increased catecholamine release and heightened sympathetic nervous system activity. In contrast, RAS is linked to increased renin levels due to renal artery narrowing [8]. A 24-hour urine test measuring metanephrines and catecholamines is crucial for distinguishing between these two conditions. Pheochromocytoma is associated with hypertension in 0.1% of the population; therefore, although this must be considered amongst possible diagnoses, the probability of this disease is low [8].

Acute nephritic syndrome shares symptoms with RAS, including hypertension, leg edema, and proteinuria. However, it usually involves high levels of proteinuria, whilst RAS presents with little to moderate proteinuria [3,9]. Additionally, nephrotic syndrome often features hematuria and glomerular inflammation, which are not characteristic of RAS [3,9].

Regarding glucocorticoid-induced HE, excessive use of glucocorticoids, along with certain medications like NSAIDs, atypical antipsychotics, and estrogen, can lead to elevated blood pressure [10]. Evaluating a patient’s medication history is essential, as these substances can contribute to or mimic the hypertension seen in RAS. Prolonged glucocorticoid use has been associated with marginal increases in blood pressure over time [10]. In summary, accurately diagnosing RAS requires careful differentiation from these other conditions, taking into account clinical presentation, laboratory tests, and medication history.

Of these differentials, hyperaldosteronism and pheochromocytoma are essential must-not-miss in addition to RAS. Our understanding of hyperaldosteronism has been significantly improved with technological advancements that go beyond the scope of assessing renin and aldosterone levels. The development of specific antibodies against human CYP11B2 (aldosterone synthase) is present within the pathology reports of resected adrenals due to primary hyperaldosteronism [11]. This gives clinicians a more definite confirmation to either rule in or rule out primary hyperaldosteronism in a patient who presents with resistant hypertension or an emergency [11]. Regarding pheochromocytoma, which can cause elevated blood pressure, it originates from different mechanisms.

Furthermore, new guidelines for pheochromocytoma have been expressed. The latest discovery in gene sequencing, circulating DNA, and circulating tumor cells helps support the pathogenesis of this tumor more so than the standard workup for pheochromocytomas [12]. Although these pathologies do exist, further workup and consideration of alternate diagnoses are necessary.

In this particular case, Doppler imaging did not demonstrate RAS. So clinical intuition had to be used alongside weighing in of pretest probability based on findings. As per the 2017 ACC/AHA clinical guidelines, evaluation of high blood pressure (due to RAS or other pathology) calls for a thorough clinical history of the patient's blood pressures over time, lifestyle, family history, medication, previous medical history, and current symptomatology. Broad diagnostic testing includes fasting blood glucose, CBC, lipid profile, renal function tests, and an electrocardiogram. Physical exams specific to RAS as the underlying pathology include stethoscope auscultation for renal and abdominal aortic bruits, along with palpation for abdominal masses and extremity pulse abnormalities to rule out additional aortorenal pathologies [13]. Systolic-diastolic abdominal bruits heard with auscultation have a specificity of 99% and sensitivity of 39% for RAS [14]. Abdominal bruits have reported likelihood ratios of 39 in systolic-diastolic abdominal bruits and range from 4.3 in systolic-only bruits [15]. High serum creatinine level, coronary artery disease severity, coronary artery bypass surgery history, congestive heart failure, pulmonary edema, and atrial fibrillation are powerful predictors of RAS [16]. Krijnen et al. found that age, sex, atherosclerotic vascular disease, recent onset hypertension, smoking history, BMI, abdominal bruit, serum creatinine, and serum cholesterol, when applied to a regression model, yielded diagnostic accuracy of RAS with a sensitivity of 72% and a specificity of 90% [17]. Thus, emphasizing the importance of a physician's adequate medical history and appropriate physical exam. Cardiovascular physical exams and extensive lab work are of paramount importance to differential formulation. These clinical variables can help a diagnosing physician understand the pretest probability for RAS in the case of suspicious imaging results, such as this case.

However, it is imperative to understand the imaging modalities, as imaging can be markedly sensitive and specific for certain presentations and further delineate diagnoses in appropriate patient presentations (Table 3). Several options are available for the diagnosis of RAS, with the gold standard being renal arteriography with intra-arterial contrast delivered through vessel catheterization, which allows the visualization of renal vessels and definitive diagnosis [3]. While renal arteriography may be highly accurate, it carries risks such as radiation exposure, arterial access, and potential adverse reactions to the contrast dye [2,3].

**Table 3:** Imaging techniques with listed sensitivity and specificity, advantages, and drawbacks CT: computed tomography, MRA: magnetic resonance angiography, IV: intravenous, RAS: renal artery stenosis

Technique	Sensitivity, specificity	Advantages	Drawbacks
Renal arteriography	Gold standard reference	Highly accurate, treatment at the time of diagnostics	Arterial access, atheroemboli, IV contrast risk
Duplex Doppler ultrasound with contrast	(94, 88)	Cost-effective, minimally invasive	Requires a skilled technician
Duplex Doppler ultrasound without contrast	(85, 79)	Cost-effective, noninvasive	Requires a skilled technician, IV contrast risk
CT angiography	(94, 93)	Multiplanar imaging and noninvasive	IV contrast risk, ionizing radiation
MRA with gadolinium contrast	(96, 93)	Minimally invasive, high-quality images	Variable accuracy in the literature, gadolinium retention
MRA without contrast	(92, 93.5)	Noninvasive, high-quality images	Variable accuracy in the literature
Captopril renal scintigraphy	(74, 59)	Can assess perfusion function over time	Not accurate in asymptomatic patients and bilateral RAS, radiation exposure

Alternative diagnostic tests also exist and include the following:

Duplex Doppler ultrasonography is a noninvasive test that visualizes the main renal arteries and measures peak systolic flow velocity in the stenotic renal artery compared to the aorta. An increased systolic velocity in the stenotic artery suggests greater stenosis, aiding in RAS diagnosis [2]. Ultrasonography is an inexpensive alternative to renal arteriography, which can be time- and resource-consuming and may present challenges in certain patients [5]. Non-contrast and contrast duplex Doppler have 85% and 79% and 94% and 88% sensitivity and specificity, respectively [18].

Computed tomographic angiography is an imaging modality that involves injecting IV contrast to visualize vessels on CT imaging and is less invasive than renal arteriography. It is highly accurate for diagnosing RAS due to atherosclerosis but may be less effective for detecting flow changes [2,3,19]. Zhang et al. The reported median sensitivity and specificity for CTA are 94% and 93%, respectively [19].

Magnetic resonance angiography (MRA) provides another noninvasive option for renal vessel imaging; however, it has shown variable sensitivity and specificity for RAS [5,19]. Non-contrast MRA has a 92% and 93.5% sensitivity and specificity, respectively, and gadolinium-enhanced MRA has a 96% and 93% sensitivity and specificity, respectively [19].

Regarding captopril renal scintigraphy, nuclear medicine studies utilize captopril and a radionuclide tracer to analyze the flow through the kidneys. Rather than being a visualization technique, this technology assesses the functionality of the kidneys [3]. Patients with bilateral stenosis and without clinical features of renovascular disease are poor candidates for this technique. Hout et al. reported a mean sensitivity of 74% and specificity of 59% for renal scintigraphy [20]. While noninvasive tests like MRA and CTA are generally safer than renal arteriography, they may be less reliable for diagnosing RAS. In summary, while noninvasive tests offer benefits in terms of patient safety and provide a helpful alternative, renal arteriography remains the gold standard for diagnosing RAS.

## Conclusions

This case underscores the importance of a thorough clinical evaluation in diagnosing RAS, especially in high-risk patients with multiple comorbidities. Although the duplex ultrasound was negative, the patient’s presentation with flash pulmonary edema and high pretest probability prompted further evaluation with renal angiography, which confirmed classic RAS. Her renal function improved significantly after angioplasty and stenting, illustrating the value of clinical judgment over sole reliance on imaging. Per the 2017 ACC/AHA guidelines and the 2022 AHA update, revascularization is indicated in cases of resistant hypertension, failed medical management, FMD, progressive renal dysfunction, and flash pulmonary edema. Incorporating physical exams, laboratory studies, and clinical context alongside imaging enhances diagnostic accuracy and reduces unnecessary resource use.
